# Psychometric assessment of the Generic Conspiracist Beliefs Scale

**DOI:** 10.1371/journal.pone.0230365

**Published:** 2020-03-19

**Authors:** Kenneth Graham Drinkwater, Neil Dagnall, Andrew Denovan, Nick Neave

**Affiliations:** 1 Department of Psychology, Faculty of Health, Psychology and Social Care, Manchester Metropolitan University, Manchester, United Kingdom; 2 Department of Psychology, Faculty of Health and Life Sciences, Northumbria University, Newcastle upon Tyne, United Kingdom; Universitat Wien, AUSTRIA

## Abstract

The Generic Conspiracist Beliefs Scale (GCBS) is the most widely used measure of general belief in conspiracy theories. The scale comprises five related but distinct factors (Government Malfeasance, Extraterrestrial Cover-up, Malevolent Global Conspiracies, Personal Wellbeing, and Control of Information). Despite this, investigators have typically treated the GCBS as unidimensional by referencing only overall total. Although, the GCBS possesses established psychometric properties, critics question its factorial structure, suggest alternative models, and recommend routine examination of GCBS dimensions as part of analysis. Through two studies, the present paper assessed GCBS factorial structure, internal reliability, convergent validity, and invariance. This involved comparing the original five-factor solution with alternative one, two, and three-factor models. To ensure that the best fitting model was robust, the authors conducted analysis in two independent samples (Study one, *N* = 794, UK university-based sample; and Study two, *N* = 418, UK respondents collected via a market research company). Results in both studies indicated superior fit for the correlated five-factor solution. This solution demonstrated invariance across gender, and samples (Study one and two). Furthermore, the total scale and five subfactors evinced good alpha and omega total reliability. Convergent validity testing exhibited associations of an expected strength between conspiracist beliefs, reality testing, and cognitive insight. Large intercorrelations existed among GCBS subfactors, suggesting that the measure reflects a narrow set of interrelated conspiracist assumptions. These findings support the use of overall scale scores as an index of belief in conspiracy theories.

## Introduction

Although the term ‘conspiracy theory’ has no single, agreed definition, conceptual delimitations share core characteristics [1]. Thematically, these centre on the belief that powerful, multiple actors manipulate events/actions in order to achieve malevolent goals [2]. A further key feature of conspiracy theories is that their narratives, despite lacking a robust evidential basis, usually cite supporting scientific evidence [3]. Accordingly, conspiracy theories can provide apparently, credible (reasoned) alternatives to mainstream, official explanations [4–6]. Conspiracy theories become particularly compelling when either an authorised account appears inadequate, or an event has no definitive explanation [7–8].

In this context, individuals engage with conspiracies because they facilitate comprehension of the origins and consequences of significant novel, or threatening events [9].

Thus, despite being dismissed by critics as fallacies [10], fringe notions (lay beliefs), and simplified views of the social and political world [11], conspiracy theories are regularly validated by members of the general population. Illustratively, in a nationally representative survey of U.S. adults, 55% of respondents endorsed at least one of several presented conspiracy theories (e.g., “The U.S. invasion of Iraq was not part of a campaign to fight terrorism, but was driven by oil companies and Jews in the U.S. and Israel”) [12]. Likewise, significant numbers of U.S. and U.K. citizens believe that their respective governments assisted or distorted information about terrorist attacks (i.e., 9/11 and 7/7 respectively) [13].

Conspiracy theories endure largely because they are resistant to falsification. Explicitly, it is difficult for detractors to establish that alleged covert activities/actions did not occur [14–15]. Furthermore, in response to disconfirming evidence conspiracy believers characteristically add layers of intrigue to legitimise theories [16]. A related issue that hinders dismissal of conspiracies is that theories are not always false (e.g., the Watergate scandal, which involved a series of intertwining illegal political actions overseen by the administration of U.S. President Richard Nixon). For believers, such exceptional instances provide justification for the authenticity of conspiratorial accounts, regardless of their inherent veracity and the implausibility of conspiracy theories generally.

Noting the societal and political importance of conspiracy theories within modern-day culture, academic interest in the topic has flourished over the past decade. Researchers contend that psychological understanding is essential because conspiratorial narratives can influence/bias individual and collective perceptions of important current (e.g., vaccinations) and historical events (e.g., moon landings). Notable negative consequences of belief in conspiracy theories are reduced involvement with and faith in social institutions (e.g., democratic, governmental and security systems) [17].

### Conceptualising conspiracy

Conspiratorial ideation refers to the proclivity to believe that clandestine groups and organisations secretly manipulate events and power relations [18]. These key refrains are central to theoretical delineations of conspiracy theories. For instance, Sunstein and Vermeule [1] define conspiracy theories as attempts to attribute outcomes to the scheming of powerful individuals, who attempt to conceal their involvement and activities. From these conceptualisations, it is clear that conspiratorial ideation embodies the canonical themes of secrecy, subterfuge and manipulation.

Congruent with the negative connotations of these features, academic theorists frequently regard conspiratorial ideation deleteriously. Concomitantly, much research explores relationships between conspiracism, the tendency to engage in conspiratorial ideation and endorse related theories, and maladaptive outcomes (e.g., cognitive-perceptual distortions, ill-rational thought processes and inaccurate world-view) [19–22].

The distinction between broad conspiratorial thinking and belief in specific conspiracy theories is important to note. Primarily, because the interchangeable use of the term obscures the important theoretical nuance that conspiratorial ideation (generic) is likely to result in endorsement of a range of theories, whereas belief in specific theories may be restricted to particular accounts. The former denotes an overarching worldview, whilst the latter is selective and focuses on particular instances. This distinction influences not only perceptions of conspiratorial theory, but also informs development of measurement tools (discussed later).

Drawing on the ideational perspective, scholars assume that conspiratorial thinking results in extreme reactions arising from an over-reliance on subjective-emotional factors and/or truncated or illogical reasoning [19, 23–24]. This perception, although principally valid, masks adaptive aspects of conspiratorial ideation. Particularly, the desire to seek truth and strive for social advancement and equity [20]. Measures of conspiracy tend to overlook potentially positive aspects of conspiratorial thinking and prefer to focus on adverse consequences.

The maladaptive viewpoint of conspiratorial ideation assumes that endorsement of theories characterises a self-protecting defence mechanism that reconciles discord between internal representations (beliefs) and conflicting external, real-world evidence (facts). This explicates why much research reports associations between conspiratorial ideation and ill-rational, unsystematic thought processes and an over-reliance on anecdotal data.

Commensurate with this view, literature examining conspiracy endorsers portrays them as suspicious, worried about personal danger, ideologically eccentric, inclined to perceiving agency in actions, and likely to treat nonsense as meaningful [25]. Hart and Graether [25] testing the validity of this generalised profile, found that bullshit receptivity (the readiness to ascribe meaning to material that implies but actually contains no sense), dangerous-world beliefs, and schizotypy both independently and additively predicted belief in conspiracies. Analysis suggested also that political orientation and hyperactive agency detection (readiness to ascribe events in the environment to the behaviour of agents) were potentially important factors.

### Measurement of conspiracy theories

It is important to acknowledge these prevailing perceptions of conspiratorial belief because they have directly informed the development of self-report instruments (i.e., content and emphasis) and the direction of research. In the case of belief in conspiracies theories, this has resulted in the production of a number of self-report scales. Swami, Barron, Weis, Voracek, Stieger, and Furnham [26] place these into two broad categories, comprising endorsement of specific conspiracy theories and validation of generic conspiracy-related beliefs. The former approach requires participants to indicate the degree to which they endorse subsets of real-world conspiracy theories (e.g., the US government orchestrated 9/11). Hence, conspiracy selection is arbitrary and bounded by investigator preference. The notion underlying this method is that substantiation of explicit theories reflects conspiratorial belief generally [8]. This perspective assumes that overall scores provide an accurate estimation of belief in conspiracy theories.

Examples include the Belief in Conspiracy Theories Inventory (BCTI; [22]), the Belief in Specific Conspiracies Scale (BSCS; [27]), and the Composite Conspiracy Beliefs Scales (CCBS; [28]) (see [26] for a detailed list of measures). These scales index a broad range of prominent theories (assassination of John F. Kennedy, government cover-up of alien landings, oil companies influencing the political decision to go to war in Iraq, etc.). Despite common application, this approach possesses limitations. Particularly, it compromises validity by sampling limited construct breadth, and overlooks factors (such as historical context, geographical location and familiarity) that influence awareness and validation of particular conspiracy theories.

Observing these issues, Brotherton, French, and Pickering [29] developed the Generic Conspiracist Beliefs Scale (GCBS), which focuses on abstract, overarching thematic concepts without reference to particular theories. For example, the notion that government agencies routinely conceal information in order to deceive the public. This idea is applicable to myriad assassination-based conspiracies (i.e., President John F. Kennedy, Princess Diana, and Osama Bin Laden), and political cover-ups (e.g., providing false details about alien visitation). This method derives from the assumption that generic belief in conspiracy theories predicts ratification of particular theories. Drawing on this idea, researchers have concomitantly developed similar measures (e.g., Conspiracy Theory Questionnaire, CTQ, [30]; the Conspiracy Mentality Questionnaire, CMB, [31]).

The present paper focused on the GCBS because it has become a recognised, widely used measure of conspiratorial belief (e.g., [32]). Indeed, researchers have translated the GCBS into several languages (i.e., French, [33]; Macedonian, [34]; Persian, [35]; Japanese [36]). Considering the effectiveness of the GCBS as an overall index of conspiratorial belief, the scale correlates highly with other widely used measures (e.g., Belief in Conspiracy Theories Inventory, BCTI, [22]; and Conspiracy Mentality Questionnaire, CMQ, [31]) [26]. Moreover, the GCBS demonstrates similar associations with criterion variables to those observed by other frequently used measures of belief in conspiracy theories. For instance, studies typically report a moderate relationship with schizotypy (see [37], GCBS; [2], BCTI; and [31], CMQ). Similarly, belief in conspiracy theories correlates moderately with belief in the paranormal. These associations are stable across a range of schizotypy and paranormal belief measures [38]. Findings indicate that the GCBS produces outcomes similar to those observed with other measures of conspiratorial belief. Overall, evidence suggests the GCBS is a conceptually sound index of belief in conspiracy theories. However, recent concerns have arisen regarding the scales factorial structure.

Brotherton et al. [29] developed the GCBS via a series of studies. Initially, participants completed 75-items reflecting broad conspiratorial notions (e.g., “Small groups of people are in possession of secret knowledge which would change our understanding of the world, and are deliberately keeping it hidden”). Exploratory factor analysis of participant responses identified five facets: Government Malfeasance (GM), Extraterrestrial Cover-up (ET), Malevolent Global Conspiracies (MG), Personal Wellbeing (PW), and Control of Information (CI). These factors subsequently informed the development of the 15-item GCBS. Ensuing studies established the reliability (internal and re-test) and validity (content, criterion-related, convergent and discriminant) of the GCBS. Relevant to the current paper confirmatory factor analysis (CFA) demonstrated that the emergent five-factor solution possessed adequate fit, and was superior to a one-factor (unidimensional) solution. Despite this, subsequent research has generally used GCBS total scores as an overall measure of belief in conspiracy theories (e.g., [30, 37]).

Notwithstanding general academic acceptance and regular use in research [38], subsequent evaluation has raised concerns about the psychometric structure of the GCBS. Explicitly, studies have failed to reproduce the five-factor model, and observed poor fit for the single factor solution (i.e., [26,35]). This suggests that the Brotherton et al. [29] model may vary as a function of sample and therefore lack measurement invariance. This is difficult to determine because studies testing factor structure are limited. However, consideration of sampling techniques across pertinent studies reveals potentially important differences.

For instance, the Brotherton et al. [29] studies recruited participants via blog, email and web, their samples were predominately British and composed of a high proportion of undergraduate students. Whereas, Swami et al. [26] used a sample of U.S. participants recruited through Amazon’s Mechanical Turk (MTurk). Utilising these data, via principal axis factor analysis (PAFA), Swami et al. [26] proposed an alternative two-factor model comprising General Conspiracist Beliefs (6-items) and Extraterrestrial Conspiracist Beliefs (4-item). Further CFA indicated poor fit for one-factor, two-factor and five-factor models.

Based on these findings, Swami et al. [26] determined that the failure to find adequate model fit was indicative of inherent problems with GCBS dimensionality. Explicitly, it was unclear whether scale latent structure indexed single or multiple dimensions of conspiratorial thinking. Consequently, Swami et al. [26] concluded that at best the scale taps into two factors, or at worst indexes multiple poorly converging dimensions. Consequently, Swami et al. [26] supported routine examination of GCBS structure as part of analysis.

Moreover, a recent Persian translation of the GCBS [35], using members from the general population from public places in Tehran, failed also to replicate the original five-factor structure. This paper was important because it drew extensively on the approach of Swami et al. [26]. Atari et al. [35], using principal-axis factor analysis, produced a novel three-factor latent structure comprising Political Conspiracies (including GM and MG), Scientific Conspiracies (combining PW and CI), and Extraterrestrial Cover-up (consistent with EC). Despite differences, this model was interpretable in the context of the Brotherton et al. [29] five-factor solution, and loaded on to a higher-order general conspiracy dimension.

Consistent with Swami et al. [26] and Atari et al. [35], Swami, Barron, Weis, and Furnham [39] reported issues with the GCBS factorial structure. Using data from UK respondents, who intended to vote in the EU membership referendum, the five- and one-factor models demonstrated poor fit. Furthermore, the Swami et al. [26] two-factor model, consisting of General Conspiracist Beliefs (GCB) and Extraterrestrial Conspiracist Beliefs, also demonstrated poor fit. Only permitting item co-variation produced acceptable fit. Based on this analysis Swami et al. [39] used only scores from the GCB factor.

### The present research

Notwithstanding criticisms, evaluation of the GCBS is limited. Hence, assessment across different samples is necessary to establish measure reliability, appropriateness and constraints [35]. This is particularly true of scale dimensionality. Noting variations in sample composition across studies assessing GCBS factorial structure, the present paper tested the applicability of the original five-factor solution within two samples often used by researchers (i.e., university-based and market research company; participation panel) [38]. The fact that it was similar in breadth and reach to that used in the validation phase of GCBS development informed inclusion of the university-based sample [see 29]. Additionally, it was representative of sampling employed by a significant proportion of studies examining belief in conspiracy theories (e.g., [8,19–20]). This assertion is based on a recent meta-analysis by Goreis and Voracek [38] of studies from the beginning of database-records until March of 2018, who found that 36.8% (N = 61) employed student samples.

The fact that recent work into conspiracy theories has utilised online participation pools such as MTurk (e.g., [40–41]) indicates that internet crowdsourcing is an important emerging method of data collection. Goreis and Voracek [38] reported that 15.7% (N = 26) of conspiracy studies used MTurk. Future numbers are likely to increase significantly because internet crowdsourcing provides samples that contain greater variation and have greater demographic diversity than traditional internet samples. Furthermore, at a practical level, online participation pools provide an accepted, expedient source of high-quality data (see [42]). Noting the trend towards using internet crowdsourcing within social science research generally, and conspiracy work specifically, the present paper assessed the fit of proposed GCBS models within a sample collected by a market research company.

Testing the factorial structure in this manner assessed the robustness of the five-factor model in a sample often used by scholars. This approach helps to identify the remit and boundaries of the GCBS. Secondly, analysis indicated whether the GCBS was an appropriate measure of general belief in conspiracy theories. This was an important question to address because the majority of psychological research has used the GCBS as an overall measure (see [38]). Accordingly, it was important to assess the fit of previously proposed factorial solutions (i.e., one, two, three and five).

The second study also tested the convergent validity of the GCBS using the known correlates proneness to reality testing deficits [8] and self-certainty [43]. Previous academic work has established that these variables positively correlate to a moderate extent with belief in conspiracy theories [8,44]. High proneness to reality testing deficits references the tendency to focus on internally generated, intra-psychic data. Accordingly, the construct is associated with a subjective-intuitive style of thinking. When this mode of thinking predominates, individuals base their perceptions of the world on personal views and feelings, inclining them to endorse conspiratorial notions [8]. Moreover, belief in conspiracies is also associated with greater self-certainty. This manifests as overconfidence in the validity of personal beliefs [43]. Hence, believers in conspiracy theories typically require less evidence before arriving at a decision and tend to demonstrate truncated logic that can result in ‘jumping-to-conclusions’ [43].

The study additionally incorporated self-reflectiveness (i.e., willingness to acknowledge the possibility of being incorrect). Researchers have not previously examined this in relation to belief in conspiracies [43], however believers in conspiracy theories typically resist alternative arguments, particularly of a rational nature [45]. Self-certainty and self-reflectiveness represent facets of cognitive insight, which can be conceptualised as the mental processes involved in self re-evaluation of anomalous experiences and misunderstandings [46–47]). Barron et al. [43] assumed that self-certainty would be the most prominent facet in relation to belief in conspiracy theories.

Inclusion of these conspiracy-related cognitive-perceptual measures enabled the authors to determine whether expected associations were consistent across GCBS subscales, or a function of particular dimensions. This was important to determine because overall GCBS scores may indicate outcomes that are actually only attributable to specific dimensions. Moreover, this analysis established whether, as suggested by Stojanov and Halberstadt [48], the presence of explicit content/actors undermines the generic nature of the GCBS. For instance, Government Malfeasance (GM) links conspiracies to governments, and Malevolent Global Conspiracies (MG) to a small group of powerful people. Finally, testing for convergent validity provided an assessment of construct validity, which is a significant criterion when testing the appropriateness of a measure [49].

Analysis also tested structural stability of the GCBS via invariance testing. Preceding research has failed to consider invariance (see [26,29,35]). Establishing invariance infers that mean differences are more likely to signify accurate mean differences rather than measurement bias [50], and is crucial in the context of measurement validation [51].

## Materials and methods

### Participants

#### Study one

Merging data sets from a previously published study [50] and progressing work produced a large sample (N = 794; *M*age = 23.26 years, *SD* = 7.90, range 18–78). There were 248 males (31%), *M*age = 25.31 years, *SD* = 9.56, range = 18–71; and 546 females (69%), *M*age = 22.35 years, *SD* = 6.82, 18–78. For all study variables, skewness and kurtosis values were within the recommended range of -2.0 to +2.0 [52]. Participant recruitment was via emails to university staff/students and local stakeholders (businesses, leisure and vocational/sports classes). If potential participants were younger than 18 years of age, or declared that they had previously taken part in similar conspiracy-based research participation discontinued. These were the only exclusion criteria. Researchers have successfully employed data amalgamation to generate large composite samples in order to evaluate scale structure and integrity [53–56].

#### Study two

A market research company (Critical Mix) was employed to recruit a large UK-based sample of adults (*N* = 418). The *M*age was 52.44 years, *SD* = 14.60, range = 18–83. The sample included 219 males (53%), *M*age = 55.90 years, *SD* = 14.07, range = 22–83; and 199 females (47%), *M*age = 48.62 years, *SD* = 14.24, range = 18–78. Skewness and kurtosis values fell between an acceptable range of -2.0 and +2.0 for all study variables.

### Measures

#### Study one

*Generic Conspiracist Beliefs Scale (GCBS)*. This study used only the 15-item GCBS. Within the measure, items appear as statements (e.g., “Secret organizations communicate with extraterrestrials, but keep this fact from the public”). Participants respond via a 5-point Likert scale (1 = definitely not true; 2 = probably not true; 3 = not sure/cannot decide; 4 = probably true; 5 = definitely true). Within the original validation paper, the GCBS demonstrated good psychometric properties) (see [29]). Specifically, validity (content, criterion-related, convergent and discriminant) and reliability (internal and re-test).

#### Study two

Alongside the GCBS, study two employed the reality testing subscale of Inventory of Personality Organization (IPO-RT; [57]) and the Beck Cognitive Insight Scale (BCIS; [46]).

*IPO-RT*. The IPO-RT assessed proneness to reality testing deficits [58–60]. Specifically, the capacity to differentiate self from non-self, intrapsychic from external stimuli, and to maintain empathy with ordinary social criteria of reality [57]. The IPO-RT adopts an information-processing approach to belief generation rather than a psychotic symptomology approach (see [61]). Noting this, authors have used the IPO-RT as an index of subjective-intuitive thinking [62].

The IPO-RT contains 20-items presented as statements (e.g., “I have seen things which do not exist in reality”). Respondents indicate their level of agreement on a five-point Likert scale, responses range from 1 = never true to 5 = always true. Total scores on the IPO-RT range from 20 to 100 with higher scores being indicative of proneness to report experiences of reality testing deficits. The IPO-RT possesses established psychometric properties. Specifically, it has demonstrated construct validity, good internal consistency and test–retest reliability indicating it is a largely psychometrically sound measure [61].

*BCIS*. The BCIS is a measure of cognitive insight, which assesses self-evaluation of judgments. The scale contains a 15-item self-report measure comprising two subscales: Self-Reflectiveness (9-items) (e.g., “I have jumped to conclusions too fast.”) and Self-Certainty (6-items) (e.g., “If something feels right, it means that it is right.”). These subscales derive from the observation that individuals with psychotic disorders (vs. psychiatric patients who did not have psychosis) are less self-reflective (e.g., reluctant to accept the possibility that they are wrong) and more assertive about their own conclusions. Researchers have used the BCIS with general samples to assess differences in critical thinking [2,63].

Items appear as statements and participants rate the extent to which they agree on a 4-point scale from 0 (do not agree at all) to 3 (agree completely). Summation of items produces subscale scores. In order to compute a composite index, it is necessary to subtract the self-certainty total from the self-reflectiveness score. The original study that validated the BCIS reported a coefficient α for the Self-Reflectiveness scale of 0.68 and for Self-Certainty 0.60 [64]. Subsequent studies reported alphas ranging from 0.72 to 0.74 for Self-Reflectiveness and from 0.72 to 0.75 for Self-Certainty [65–66]. These suggest a reasonable degree of internal consistency.

## Procedure

Respondents across studies followed the same general protocol (all projects centred on scientifically unsubstantiated beliefs, cognitive-perceptual factors and decision-making). The only procedural difference between the two studies in this paper was that participants in Study one completed measures either online or in paper form, whereas Study two was entirely online. Qualtrics hosted the internet version, which potential respondents accessed via a web link.

Prior to participation, respondents received detailed background information. This stated the nature of the study and outlined ethics. Only respondents providing informed consent progressed to provide brief demographic details (age, preferred gender and general location) and complete the scales. Prior to responding, instructions asked participants to carefully read the questions, answer all items, take their time, and respond in an open and honest manner. To prevent order effects, scale position randomly rotated across respondents.

### Ethics

For Study one, as preparation for grant bids (October 2016, 2017, and 2018) the researchers gained ethical authorisation for a series of studies examining belief in conspiracy theories and cognitive-perceptual personality factors. Following formal submission, the Director of the Research Institute for Health and Social Change and the Manchester Metropolitan University Faculty of Health, Psychology and Social Care Ethics Committee gave ethical approval.

For Study two, the research team obtained ethical authorization for the project ‘Relationships between personality and conspiracist ideation’. The study investigated the links between certain personality characteristics and belief in conspiracy theories. The Faculty of Health and Life Sciences Research Ethics Committee at Northumbria University provided ethical approval.

In order to participate in the studies respondents provided informed consent by ticking/clicking a box prior to the self-report measures indicating that they understood the nature of the study and intended to participate. Participants did this with the awareness that they could cease participation at any point during completion of the measures. Additionally, the briefing instructions informed participants of their right to withdraw submitted responses up to four weeks after completion. Withdrawal at this stage required emailing the research team with a unique personal identifier.

### Analysis

Study one, using confirmatory factor analysis (CFA), assessed the adequacy of proposed GCBS solutions. A unidimensional model operated as a baseline contrast for subsequent models. Models tested included those proposed by Brotherton et al. [29], Swami et al. [26] and Atari et al. [35]. These propose different numbers of correlated factors.

The Brotherton et al. [29] original model comprises five-factors: Government Malfeasance (GM), Extraterrestrial Cover-up (EC), Malevolent Global (MG), Personal Wellbeing (PW), and Control of Information (CI). Whereas, the two-factor condensed Swami et al. [26] model contains General Conspiracist Ideation (GC) and Conspiracist Beliefs about Extraterrestrial Life (EL). Lastly, Atari et al. [35] identified three subfactors (Political Conspiracies, PC; Scientific Conspiracies, SC; and Extraterrestrial Cover-up; ExC).

A number of indices determined goodness of fit. Specifically, the chi-square (*χ*^*2*^) statistic, relative fit (Comparative Fit Index, CFI; Tucker-Lewis Index, TLI), and absolute fit indices (Root-Mean-Square Error of Approximation, RMSEA; Standardised Root-Mean-Square Residual, SRMR). For relative indices, values > 0.90 indicate good fit [67]. For absolute indices, values of .05, .06-.08, and .08–1.0 suggest good, satisfactory, and marginal fit [68]. Consultation of RMSEA incorporated the 90% confidence interval (CI). All CFA analyses (using Mplus 7.4 [69]) employed the robust maximum likelihood (MLR) method, which yields maximum likelihood parameter estimates and standard errors that are robust to occurrences of data non-normality [70]. Akaike’s Information Criterion (AIC) compared solutions with an equal number of variables (thus, the unidimensional, five-factor and three-factor models). Lower values designate greater data-fit.

Invariance testing for Study one comprised multi-group CFA. This examined factor structure (configural), factor loadings (metric) and item intercepts (scalar) among men and women for the superior factor model. For each invariance test, a CFI difference of ≤ 0.01 and RMSEA difference of ≤ 0.015 [71] determined suitable fit. Lastly, reliability testing of the GCBS involved computing Cronbach’s alpha and omega total coefficients (using SPSS and Mplus 7.4 respectively).

Identical stages of model testing occurred for Study one and Study two to determine the replicability of the results from Study one in an independent sample. In addition, invariance testing examined the structural equivalence of the GCBS among the Study one and Study two samples. This comprised the same range of tests and model fit criteria as the gender invariance analyses. For completeness, reliability testing occurred also for IPO-RT, Total BCIS, Self-Certainty and Self-Reflectiveness. Finally, a test of convergent validity in Study two involved assessing total GCBS and subfactor correlations with suitable criterion measures. Particularly, Reality Testing (via the Reality Testing subscale of The Inventory of Personality Organization; IPO-RT), total Cognitive Insight (using the Beck Cognitive Insight Scale; BCIS), Self-Certainty and Self-Reflectiveness (via BCIS subscales).

## Results

### Study one

The undimensional model demonstrated poor fit on all indices but SRMR, *χ*^*2*^ (90, *N* = 794) = 803.431, *p* < .001, CFI = 0.857, TLI = 0.833, RMSEA = 0.100 (90% CI of 0.094 to 0.106), SRMR = 0.057. The correlated five-factor model (22) reported good fit on all indices, *χ*^*2*^ (80, *N* = 794) = 287.130, *p* < .001, CFI = 0.958, TLI = 0.946, RMSEA = 0.057 (90% CI of 0.050 to 0.064), SRMR = 0.033. The two-factor solution (18) had negative variance and unsatisfactory fit (apart from SRMR), *χ*^*2*^ (34, *N* = 794) = 490.424, *p* < .001, CFI = 0.848, TLI = 0.799, RMSEA = 0.130 (90% CI of 0.120 to 0.140), SRMR = 0.060. The three-factor model (28) produced good fit overall, *χ*^*2*^ (87, *N* = 794) = 498.171, *p* < .001, CFI = 0.918, TLI = 0.901, RMSEA = 0.077 (90% CI of 0.071 to 0.084), SRMR = 0.045.

**Fig 1:**
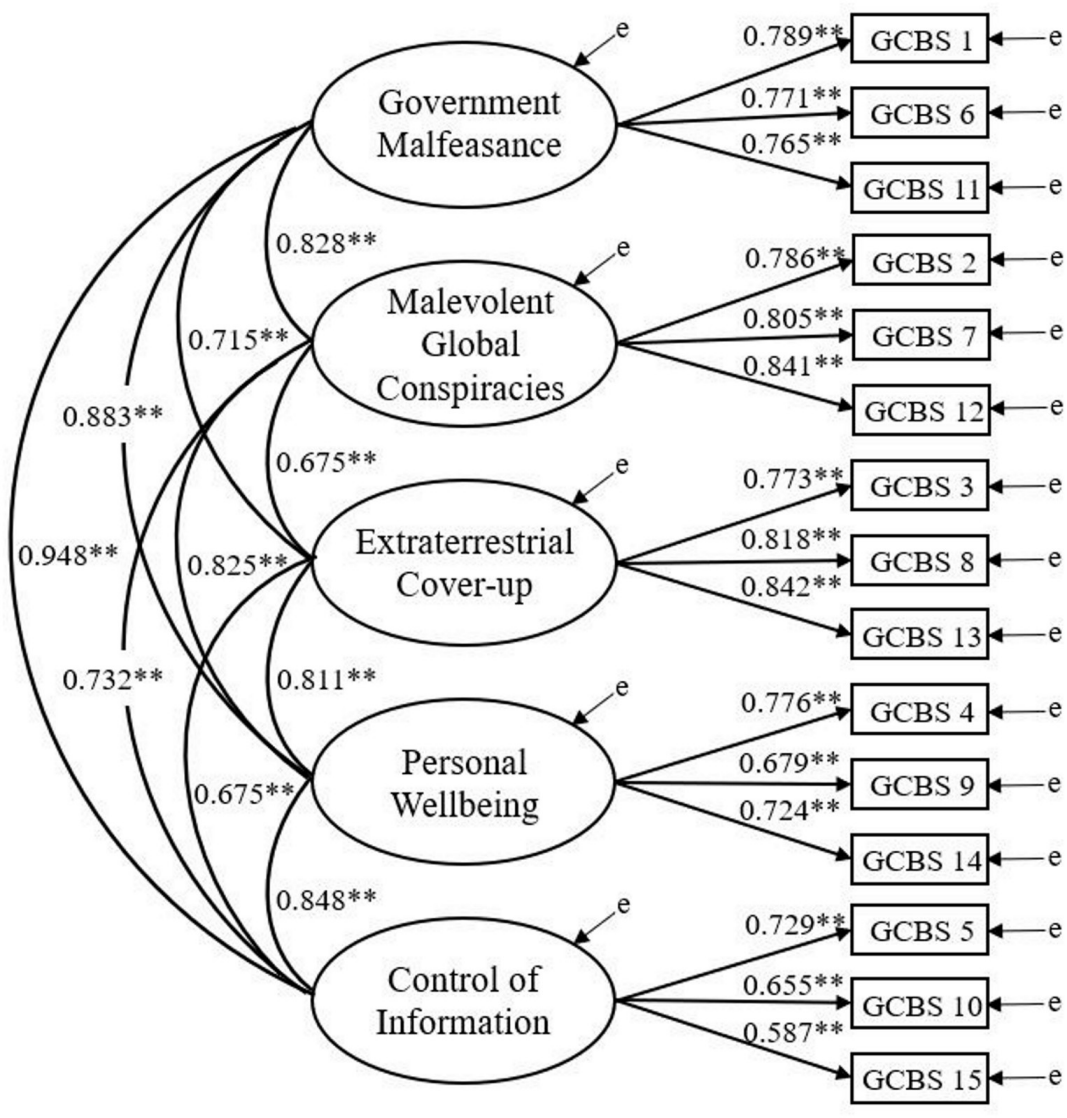
Five-factor GCBS model for Study one. Ellipses represent latent variables; rectangles represent measured variables; ‘e’ represents error. ** *p* < .001.

Comparison of AIC revealed a lower estimate for the five-factor model (31199.451) vs. the three-factor (31458.723) and unidimensional solutions (31862.623). Evaluation of fit indices and AIC indicated that the five-factor model (Fig 1) fit the data the best. Inspection of standardized parameter estimates revealed that all items (apart from item 15; loading of 0.587) loaded above the strict condition of 0.6 [71]. In addition, all five factors demonstrated large intercorrelations (Table 1).

**Table 1:** Study one and Study two intercorrelations.

Variable	*M*	*SD*	1	2	3	4	5	6	7	8	9	10
Study one												
1. Total GCBS	47.649	12.445		0.878**	0.839**	0.814**	0.869**	0.788**				
2. GM	10.233	2.934			0.692**	0.599**	0.694**	0.713**				
3. MG	9.405	3.120				0.578**	0.668**	0.556**				
4. ET	8.352	3.313					0.660**	0.514**				
5. PW	8.575	3.032						0.604**				
6. CI	11.083	2.438										
Study two												
1. Total GCBS	39.368	13.853		0.889**	0.891**	0.810**	0.911**	0.828**	0.516**	0.292**	0.253**	0.243**
2. GM	7.408	3.207			0.767 **	0.631 **	0.775**	0.677**	0.471**	0.262**	0.229**	0.214**
3. MG	8.000	3.306				0.638**	0.771**	0.674**	0.453**	0.237**	0.217**	0.180**
4. ET	6.893	3.321					0.677 **	0.541**	0.496**	0.272**	0.207**	0.265**
5. PW	7.631	3.198						0.723**	0.513**	0.261**	0.240**	0.197**
6. CI	9.434	2.959							0.290**	0.233**	0.202**	0.193**
7. IPO-RT	41.760	15.909								0.483**	0.518**	0.264**
8. Total BCIS	33.078	6.928									0.893**	0.792**
9. Self-Certainty	19.254	4.692										0.432**
10. Self-Reflectiveness	13.824	3.457										

GCBS, Generic Conspiracist Beliefs Scale; GM, Government Malfeasance; MG, Malevolent Global Conspiracies; ET, Extraterrestrial Cover-up; PW, Personal Wellbeing; CI, Control of Information; IPO-RT, Reality Testing; BCIS, Beck Cognitive Insight Scale.

** indicates *p* < .001

Invariance testing comparing men and women suggested good model fit across indices at the configural level, χ^2^ (160, *N* = 794) = 398.258, *p* < 0.001, CFI = 0.953, TLI = 0.938, RMSEA = 0.061 (90% CI of 0.054 to 0.069), SRMR = 0.038. Assessment of metric invariance reported a satisfactory CFI difference of 0.002 and no difference in RMSEA. For scalar invariance, an acceptable CFI difference of 0.003 existed with no difference in RMSEA. Results support invariance of form, factor structure and intercepts.

High internal reliability existed for total GCBS (*α* = 0.930). Likewise, subfactors of GM (*α* = 0.818), EC (*α* = 0.851), MG (*α* = 0.851), PW (*α* = 0.774), and CI (*α* = 0.699) demonstrated acceptable to good internal consistency. Omega total evinced equivalent results to alpha: high reliability for total GCBS (*ω* = 0.931), and acceptable to good reliability for all subfactors (GM *ω* = 0.819; EC *ω* = 0.852; MG *ω* = 0.852; PW *ω* = 0.771; CI *ω* = 0.700).

### Study two

The unidimensional model revealed good fit on CFI and SRMR. TLI was unsatisfactory and RMSEA was marginal, *χ*^*2*^ (90, *N* = 418) = 382.533, *p* < .001, CFI = 0.902, TLI = 0.885, RMSEA = 0.088 (90% CI of 0.079 to 0.097), SRMR = 0.050. A correlated five-factor solution suggested good fit overall, *χ*^*2*^ (80, *N* = 418) = 170.416, *p* < .001, CFI = 0.970, TLI = 0.960, RMSEA = 0.052 (90% CI of 0.041 to 0.063), SRMR = 0.031. Consistent with Study one, a test of the two-factor model revealed the presence of error variance. Good fit existed for CFI and SRMR, and unsatisfactory fit was apparent for TLI and RMSEA, *χ*^*2*^ (34, *N* = 418) = 195.447, *p* < .001, CFI = 0.905, TLI = 0.875, RMSEA = 0.106 (90% CI of 0.092 to 0.121), SRMR = 0.051. Good fit existed for the three-factor solution, *χ*^*2*^ (87, *N* = 418) = 229.592, *p* < .001, CFI = 0.952, TLI = 0.942, RMSEA = 0.062 (90% CI of 0.053 to 0.072), SRMR = 0.037.

**Fig 2:**
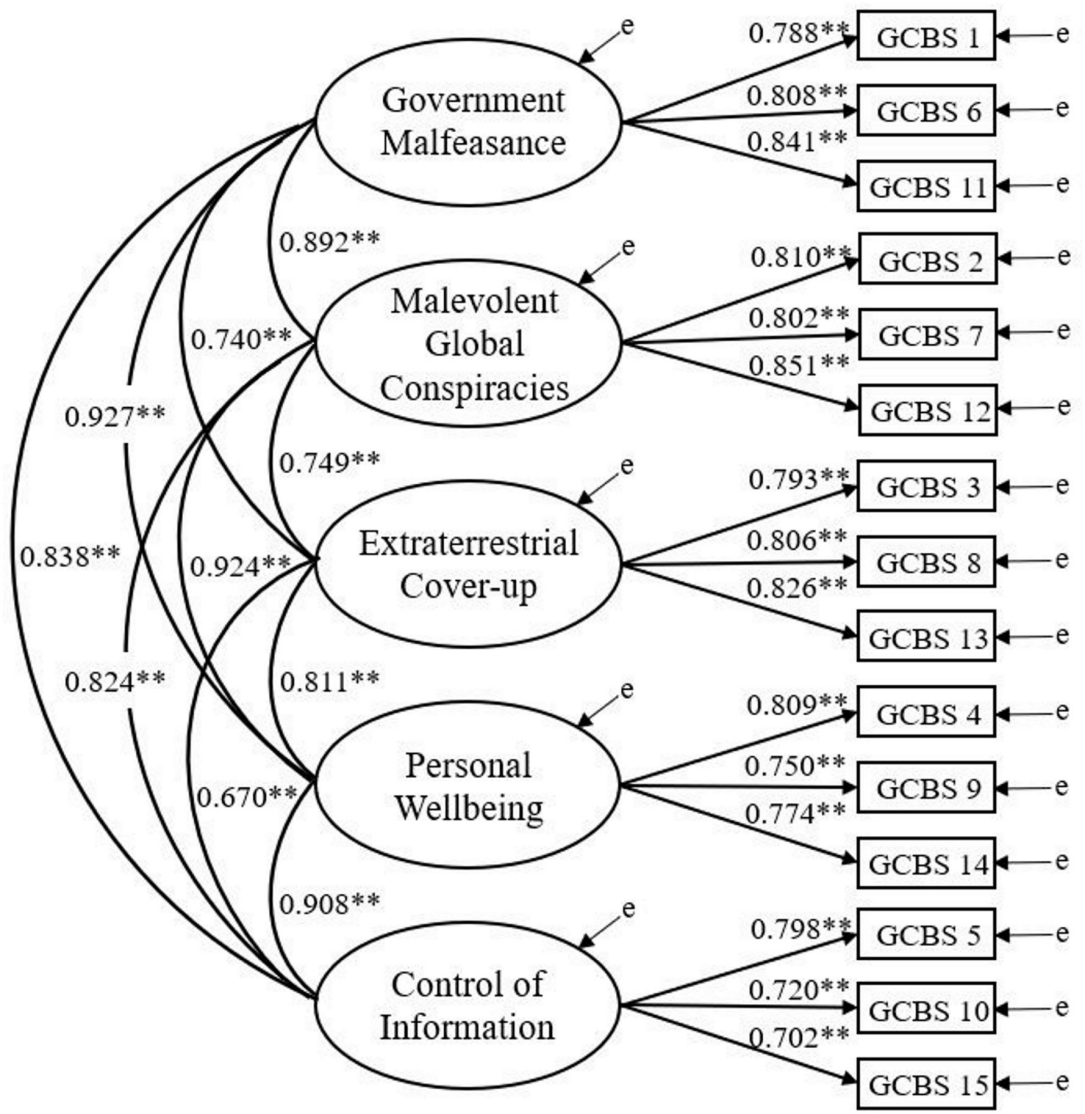
Five-factor GCBS model for Study two. Latent variables are represented by ellipses; measured variables are represented by rectangles; error is represented by ‘e’. ** p < .001.

The AIC statistic indicated a lower estimate for the five-factor model (16587.554) in comparison with the three-factor (16655.384) and unidimensional model (16864.292). Consistent with Study one, the five-factor model (Fig 2) reports superior data-model fit. Standardized parameter estimates inferred that all items exceeded 0.6. All subfactors evidenced large intercorrelations (Table 1).

Gender invariance in relation to form (configural) suggested good fit, χ^2^ (160, *N* = 418) = 258.462, *p* < 0.001, CFI = 0.968, TLI = 0.957, RMSEA = 0.054 (90% CI of 0.042 to 0.066), SRMR = 0.037. A difference of 0.001 existed for both CFI and RMSEA when examining metric invariance. At the scalar level, a satisfactory CFI difference of 0.002 was apparent, with no difference in RMSEA. Thus, the GCBS was invariant across gender.

Multi-group analysis comparing Studies one and two reported good model fit at the configural stage, χ^2^ (160, *N* = 1212) = 450.931, *p* < 0.001, CFI = 0.964, TLI = 0.952, RMSEA = 0.055 (90% CI of 0.049 to 0.061), SRMR = 0.031. At the metric level, satisfactory CFI and RMSEA differences of 0.001 existed. Scalar invariance testing reported acceptable CFI (0.007) alongside a suitable RMSEA difference of 0.003. Findings support invariance among the Study one and Study two samples.

Similar to Study one, total GCBS demonstrated high internal consistency (*α* = 0.945). Equally, good internal consistency existed for subfactors of GM (*α* = 0.854), EC (*α* = 0.861), MG (*α* = 0.849), PW (*α* = 0.821), and CI (*α* = 0.787). Omega total for total GCBS was high (*ω* = 0.934), and was good for all subfactors (GM *ω* = 0.854; EC *ω* = 0.861; MG *ω* = 0.849; PW *ω* = 0.821; CI *ω* = 0.787). Alpha and omega total were high for IPO-RT (*α* = 0.952 and *ω* = 0.953), good for Total BCIS (*α* = 0.830 and *ω* = 0.822), marginally acceptable for Self-Certainty (*α* = 0.680 and *ω* = 0.690), and good for Self-Reflectiveness (*α* = 0.823 and *ω* = 0.824).

Assessment of convergent validity revealed moderate to large positive correlations between total GCBS and GCBS subscales with Reality Testing (IPO-RT). Small positive correlations existed between total GCBS and GCBS subscales with total BCIS, Self-Certainty and Self-Reflectiveness (Table 1).

## Discussion

The present study examined the psychometric properties of the Generic Conspiracist Beliefs Scale (GCBS) [29], which is a recognised, widely used measure of belief in conspiracy theories (e.g., [32]). In this context, researchers typically assume the GCBS provides a global, unidimensional solution. This interpretation ignores alternative factorial solutions, and important conceptual concerns about GCBS content (see [26,29,35,39]. Indeed, justification for GCBS scoring extends rarely beyond a cursory citing of the original development and validation report (e.g., [23,37,72]). Acknowledging these issues, this paper evaluated the structure and measurement properties of the GCBS. This not only helps to legitimise previous findings, but also ensures that subsequent work scores the instrument appositely.

The original five-factor solution [29], comprising Government Malfeasance, Extraterrestrial Cover-up, Malevolent Global Conspiracies, Personal Wellbeing, and Control of Information, produced good data-model fit in two independent samples (i.e., university-based and market research company; participation panel). Further analysis revealed that high internal reliability (alpha and omega total) existed for full-scale and subfactor scores. Moreover, the five-factor solution demonstrated superior fit to competing models (unidimensional; two-factor [26]; and three-factor [35]).

In line with Brotherton et al. [29], large intercorrelations existed between factors. This finding was consistent with the view that GCBS dimensions reflect associated assumptions. The presence of related factors provides some justification for using the full-scale score as a global index of belief in conspiracy theories. From a practical perceptive, this also provides a rationale for employing the GCBS as a brief, expedient measure.

Study two, where correlations between GCBS subfactors were generally consistent across convergent measures, provided further vindication for the use of the full-scale score. In the case of BCIS, all correlations across Self-Certainty and Self-Reflectiveness dimensions were in the small (*r* = .10) to medium (*r* = .30) range [73]. Concerning IPO-RT, the correlation with Control of Information (*r* = .29) was weaker than associations with Government Malfeasance, Malevolent Global Conspiracies, Extraterrestrial Cover-up, and Personal Wellbeing (these ranged from *r* = .45 to .52).

This outcome is difficult to interpret. Looking at Control of information, the subfactor indexes unethical control and suppression of information by organizations, including the government, the media, scientists and corporations. At face value, this outcome tentatively suggests that notions of scientific concealment and technological manipulation are less intuitively appealing. Clearly, further work in this domain is required.

GCBS full-scale scores correlated weakly with BCIS dimensions Self-Certainty and Self-Reflectiveness, and moderately with the IPO-RT. Self-certainty findings concurred with those of Barron et al. [43], who found a moderate positive correlation between the factor and endorsement of conspiracist beliefs (Belief in Conspiracy Theories Inventory, BCTI, [22]). Existence of a weaker correlation between self-reflectiveness and GCBS scores compared to self-certainty and GCBS scores supports the view that self-reflectiveness would be less prominent among believers in conspiracy theories [43]. Additionally, the positive correlation between IPO-RT aligned with that reported by Drinkwater et al. [8]. Collectively, these results concur with the view that belief in conspiracy theories is concomitant with overconfidence in the validity of personal beliefs [43] and higher levels of subjective-intuitive thinking [see 50].

Support for the five-factor GCBS solution did not accord with recent work by Swami et al. [26, 39] and Atari et al. [35]. Looking at the composition of their samples (i.e., crowd sourcing and general population from public places) and comparing them to the present paper (i.e., university-based and market research provider), there were no obvious or systematic differences that could explain the observed variations in factorial structure. Furthermore, articles producing and replicating the five-factor solution have employed a range of recruitment techniques (i.e., blog post, online forums, and emailing list) [22]. These sampling approaches are representative of self-report studies investigating belief in conspiracy theories generally (see [38]).

Within the current paper, the GCBS demonstrated invariance of form, factor structure and intercepts across gender in both independent samples. Moreover, satisfactory invariance existed when comparing across the UK-based samples (i.e., studies one and two). Overall, consideration of present and previous work suggests that the GCBS five-factor structure is not an artefact arising from a particular sample type. Although, further research into potential measurement bias is required since the original validation and present validation studies were UK-focused, whereas Swami et al. [26] and Atari et al. [35] used samples drawn from the USA and Iran respectively.

Noting this, subsequent work should examine scale invariance across wider ranging contexts in order to delimit situations where the five-factor and one-factor solutions are most appropriate. This includes extensive cross-cultural comparisons because variations in societal norms are likely to influence belief in conspiracy theories. A recent example of this is the Japanese Version of the Generic Conspiracist Beliefs Scale (GCBS-J) [36], which supported a two-factor structure comprising General Conspiracist Beliefs and Extraterrestrial Conspiracist Beliefs. Tellingly, the authors suggested this two-factor structure emerged due to differences in the nature of conspiracy beliefs among Japanese vs. Western societies. Widespread cross-cultural comparison would extend the process initiated by the present paper with British samples to other countries. An additional academic benefit is that this comparison will reveal cultural differences in prevalence and content of conspiracy theories.

The observation that the factorial structure of the GCBS may be prone to contextual variation is consistent with Bruder et al. [31], who contend that the use of specific content-related detail gives rise to cultural response variations. This issue pertains to the GCBS because it contains items that refer to explicit topics, such as technology (i.e., “New and advanced technology which would harm current industry is being suppressed”) and terrorism (i.e., “The government permits or perpetrates acts of terrorism on its own soil, disguising its involvement”). This specificity introduces the potential for contextual bias.

For instance, views of technological advance vary across societies. Democratic countries generally regard technological advance as progressive and financially necessary, whereas autocratic states often frustrate technical development for political/economic reasons. Similarly, social, religious and geographical factors can influence perceptions of terrorism. Illustratively, it is common knowledge that the U.S. government planned a false flag operation (Operation Northwoods) in 1962 [74]. This recommended staging an attack on American soil in order to provide a justification for attacking Cuba. In this instance, individuals with awareness of Operation Northwoods, who endorse the notion of orchestrated terrorist attacks, are indicating political and historical awareness rather than belief in conspiracy theories. This point concurs with Stojanov and Halberstadt [48], who contend that the presence of factors that refer to explicit conspirators (i.e., government and powerful people) may undermine the generic nature of the GCBS.

The inclusion of thematic specificity within the GCBS also introduces possible temporal instability. This arises from the fact that belief in particular theories changes over time. Some theories increase in popularity, whereas others decline. In the case of terrorism for instance, awareness of false flag operations fluctuates because of media attention. Tentatively, this may explain the structural variations observed by Swami et al. [26,39]. Future studies could examine this by comparing item endorsement across multiple time points. Such repeated test-retest would establish the extent to which factors and items possess temporal stability. These criticisms suggest that the measurement of belief in conspiracy theories benefits from adopting a focus on overarching thematic ideology and concepts. From this perspective, the multidimensional GCBS is better suited for exploring domain-specific differences in conspiracy beliefs. The extent to which these are generalizable depends on ensuing work establishing scale invariance.

The issue of factorial stability is not unique to the GCBS. Other psychometric instruments experience similar difficulties. For example, questions concerning stability exist for the Mental Toughness Questionnaire 48 (MTQ48, [75]), which is a measure of the capability to cope with difficulties and to achieve self-defined aims [76–77]. The scale authors report that four high-order dimensions (i.e., 4Cs: Challenge, Commitment, Control and Confidence) exist. Perry, Clough, Crust, Earle, and Nicholls [78] provided support for this solution. However, other researchers have reported large degrees of misspecification with samples comprised of elite, amateur and non-athletes [79]. Other studies have also failed to reproduce the 4C solution, and questioned its appropriateness [77,80]. Explicitly, Gucciardi et al. [80] was unable to demonstrate good data fit in athlete and workplace samples.

Finally, subsequent studies could attempt to explain observed variations in GCBS factor structure by testing the various models in a large, heterogeneous sample. Investigators could achieve this by aggregating publicly available data. Increasing sample size will reduce the potential influence of random factors, and should result in the production of a purified factorial structure. Ultimately, this may result in the modification of existing items and recommend the generation of new questions. This process, consistent with points raised in this paper will facilitate the further development of generic, culture free content.
